# Surgery Versus Stereotactic Body Radiotherapy for Early-Stage Non-small Cell Lung Cancer (NSCLC): A Comprehensive Review of Survival and Local Control Outcomes

**DOI:** 10.7759/cureus.84440

**Published:** 2025-05-19

**Authors:** Abdulrahman Bin Sumaida, Nandan M Shanbhag, Nadeem Pervez, Khalifa AlKaabi, Khalid Balaraj

**Affiliations:** 1 Oncology/Radiation Oncolgy, Tawam Hospital, Al Ain, ARE; 2 Radiation Oncology/Palliative Care, Tawam Hospital, Al Ain, ARE; 3 Oncology, University of Alberta, Alberta, CAN; 4 Oncology, Tawam Hospital, Al Ain, ARE; 5 Department of Internal Medicine, College of Medicine and Health Sciences, United Arab Emirates University, Al Ain, ARE; 6 Radiation Oncology, Tawam Hospital, Al Ain, ARE

**Keywords:** lobectomy, meta-analysis, non-small cell lung cancer, sabr, sbrt, stereotactic body radiotherapy, surgery

## Abstract

This systematic review and meta-analysis aimed to compare the efficacy of stereotactic body radiotherapy (SBRT) and surgical resection in early-stage non-small cell lung cancer (NSCLC), focusing on overall survival (OS), cancer-specific survival (CSS), and local control (LC). A comprehensive literature search was conducted using PubMed, Cochrane Library, ScienceDirect, and Google Scholar, and eligible studies were selected according to PRISMA guidelines. Pooled hazard ratios (HRs) with 95% confidence intervals (CIs) were calculated for OS, CSS, and LC using fixed- or random-effects models, and the Newcastle-Ottawa Scale was used to assess study quality. A total of 41 studies involving 88,228 patients (58,366 treated with surgery and 29,862 with SBRT) were included. Surgical resection was significantly associated with improved three-year OS (HR = 1.39; 95% CI: 1.25-1.55; p < 0.00001) compared to SBRT. Subgroup analysis revealed greater survival benefits with lobectomy (HR = 1.50; p < 0.00001) than sublobar resection (HR = 1.27; p = 0.002) or mixed approaches (HR = 1.39; p = 0.007). CSS also favored surgery (HR = 1.22; p = 0.006), particularly lobectomy (HR = 1.46; p = 0.002). LC was comparable between SBRT and surgery (HR = 0.92; p = 0.06), although lobectomy showed a slight advantage (HR = 0.92; p = 0.04). These findings suggest that surgical resection, especially lobectomy, offers superior OS and CSS compared to SBRT in early-stage NSCLC, while LC outcomes are generally equivalent. SBRT remains an effective alternative for medically inoperable patients; however, in operable candidates, surgery should be considered the preferred approach to maximize long-term outcomes.

## Introduction and background

Lung cancer is the leading cause of cancer-related mortality worldwide, accounting for an estimated 1.8 million deaths in 2020 alone [1,2]. Non-small cell lung cancer (NSCLC) constitutes approximately 85% of all lung cancer diagnoses [3,4]. When detected at an early stage, typically stage I or II, NSCLC is potentially curable, making timely intervention critical. However, many patients are diagnosed late due to the asymptomatic nature of early disease and the lack of effective screening protocols globally. With increasing implementation of low-dose computed tomography (LDCT) screening in high-risk populations, early detection rates have improved, enhancing the prospects for curative treatment [5].

Surgical resection, most commonly lobectomy with systematic mediastinal lymph node dissection or sampling, has long been considered the gold standard for treating operable early-stage NSCLC, offering five-year overall survival (OS) rates of approximately 70-90% in appropriately selected patients [6]. However, a substantial proportion of patients are deemed medically inoperable due to advanced age, poor pulmonary reserve, or significant comorbidities [7,8]. Stereotactic body radiotherapy (SBRT), also referred to as stereotactic ablative radiotherapy (SABR), has emerged as a viable non-invasive alternative. SBRT delivers high doses of radiation in a limited number of fractions with sub-millimeter precision, minimizing exposure to surrounding healthy tissues [9,10]. Initial studies demonstrated excellent local control rates with SBRT, ranging from 87% to 97.8% at two years, even in high-risk surgical candidates [11]. Consequently, SBRT has become the standard of care for patients with inoperable early-stage NSCLC, and its role is now being explored in operable populations as well [12]. Despite promising results, uncertainty persists regarding its comparative long-term outcomes, especially in terms of overall survival and disease recurrence, when weighed against surgical resection.

Despite SBRT's increasing adoption and promising outcomes, the optimal treatment for operable early-stage NSCLC remains a subject of ongoing debate. Surgery provides the opportunity for pathological staging and potential removal of occult nodal disease, which SBRT does not [13]. However, SBRT offers a non-invasive alternative with minimal perioperative risk, an important consideration for elderly patients or those with compromised cardiopulmonary function [14]. Some retrospective studies and pooled analyses have suggested comparable overall survival between SBRT and surgery in selected operable patients, particularly when perioperative mortality is accounted for [15,16]. 

Several systematic reviews and meta-analyses have evaluated the comparative effectiveness of SBRT and surgery in early-stage NSCLC, often drawing from observational or propensity score-matched studies. While earlier reviews highlighted comparable local control and cancer-specific survival, they also acknowledged higher overall survival with surgery, possibly reflecting differences in baseline health status rather than true treatment superiority [16-18]. However, the landscape of lung cancer treatment has evolved. Advances in SBRT delivery (e.g., image-guided adaptive planning), better patient selection through multidisciplinary evaluation, and longer follow-up data from both surgical and SBRT cohorts now allow for a more informed comparison. Given these developments and the inclusion of newly published high-quality studies, an updated synthesis of the evidence is warranted to reassess the balance between efficacy and risk in both modalities. This systematic review and meta-analysis aims to compare the efficacy of SBRT/SABR versus surgery in patients with early-stage NSCLC.

## Review

Methods

This systematic review and meta-analysis were conducted following the PRISMA (Preferred Reporting Items for Systematic Reviews and Meta-Analyses) recommendations [19].

Data Sources and Search Strategy

A systematic literature search was done in PubMed, Cochrane Library, ScienceDirect, and Google Scholar, for articles published from database inception up to April 1, 2025 (Appendix 1). The keywords used were "non-small cell lung carcinoma", "non-small cell lung cancer", "lung adenocarcinoma", "lung squamous cell carcinoma", "large cell lung cancer", "surgery", "lobectomy", "sublobar resection", "limited resection", "sublobectomy", "segmentectomy", "wedge resection", "stereotactic ablative radiotherapy", "stereotactic body radiotherapy", "SBRT”, and "SABR". Boolean operators OR and AND were used to develop the search strings. The initially identified articles were then subjected to a study selection procedure.

Inclusion and Exclusion Criteria

Studies were included if they met the following criteria: (1) adult patients (≥18 years) diagnosed with early-stage NSCLC, defined as stage I or tumors classified as T1 to T3N0, without regional lymph node involvement; (2) studies examining patients treated with SBRT/SABR; (3) comparison studies between SBRT and surgery; (4) studies had to report on at least one of the following outcomes: OS, cancer-specific survival (CSS), or local control (LC); (5) randomized controlled trials (RCTs), prospective or retrospective cohort studies, and comparative observational studies that employed adjustment for confounding (e.g., propensity score matching or multivariable regression); (6) studies were required to report a minimum median follow-up of at least 12 months; and (7) studies published in English.

We excluded studies that did not provide a direct comparison between SBRT/SABR and surgical resection. Case reports, small case series with fewer than 10 patients per arm, letters to the editor, editorials, and conference abstracts lacking full-text publication were also excluded. In addition, we excluded narrative reviews, systematic reviews, and meta-analyses. Studies with small sample sizes, defined as fewer than 20 patients in either treatment arm, were not considered for inclusion.

Two independent reviewers screened the titles and abstracts of all identified studies. Full-text articles were retrieved for studies that appeared to meet the inclusion criteria, but there was uncertainty. Disagreements were resolved by consensus or through consultation with a third reviewer. 

Outcomes

The meta-analysis evaluated three-year outcomes for OS, CSS, and LC. Studies were excluded if they involved combined treatment modalities in either comparison group or lacked data on three-year OS, CSS, or LC.

Data Extraction and Quality Assessment

Data were independently extracted by three reviewers using a standardized data extraction MS Excel sheet (Microsoft Corporation, USA) to ensure consistency and minimize bias. The following information was collected from each included study: first author, year of publication, country, study design, clinical stage of NSCLC, number of patients (including percentage of males), SBRT radiation dose and fractionation, type of surgery performed, reported outcomes, and median duration of follow-up (in months) for both SBRT and surgical groups. Any discrepancies during data extraction were resolved by consensus or consultation with a fourth reviewer. The methodological quality of non-randomized studies was assessed using the Newcastle-Ottawa Scale, which evaluates selection, comparability, and outcome domains [20]. Potential publication bias was assessed through funnel plots.

Statistical Analysis

Meta-analyses were conducted using Review Manager software (version 5.4). For dichotomous outcomes, hazard ratios (HRs) with corresponding 95% confidence intervals (CIs) were utilized. When not directly reported, survival data were extracted from Kaplan-Meier curves following the method proposed by Tierney et al. [21]. Statistical heterogeneity across studies was assessed using the I² statistic and Cochran’s Q test. In cases where significant heterogeneity was detected under the fixed-effect model, a random-effects model was applied to produce pooled estimates. Subgroup analyses were carried out based on the type of surgical intervention (lobectomy, sublobar resection, or mixed procedures). A p-value of < 0.05 was considered statistically significant.

Results

Search Results

**Figure 1:**
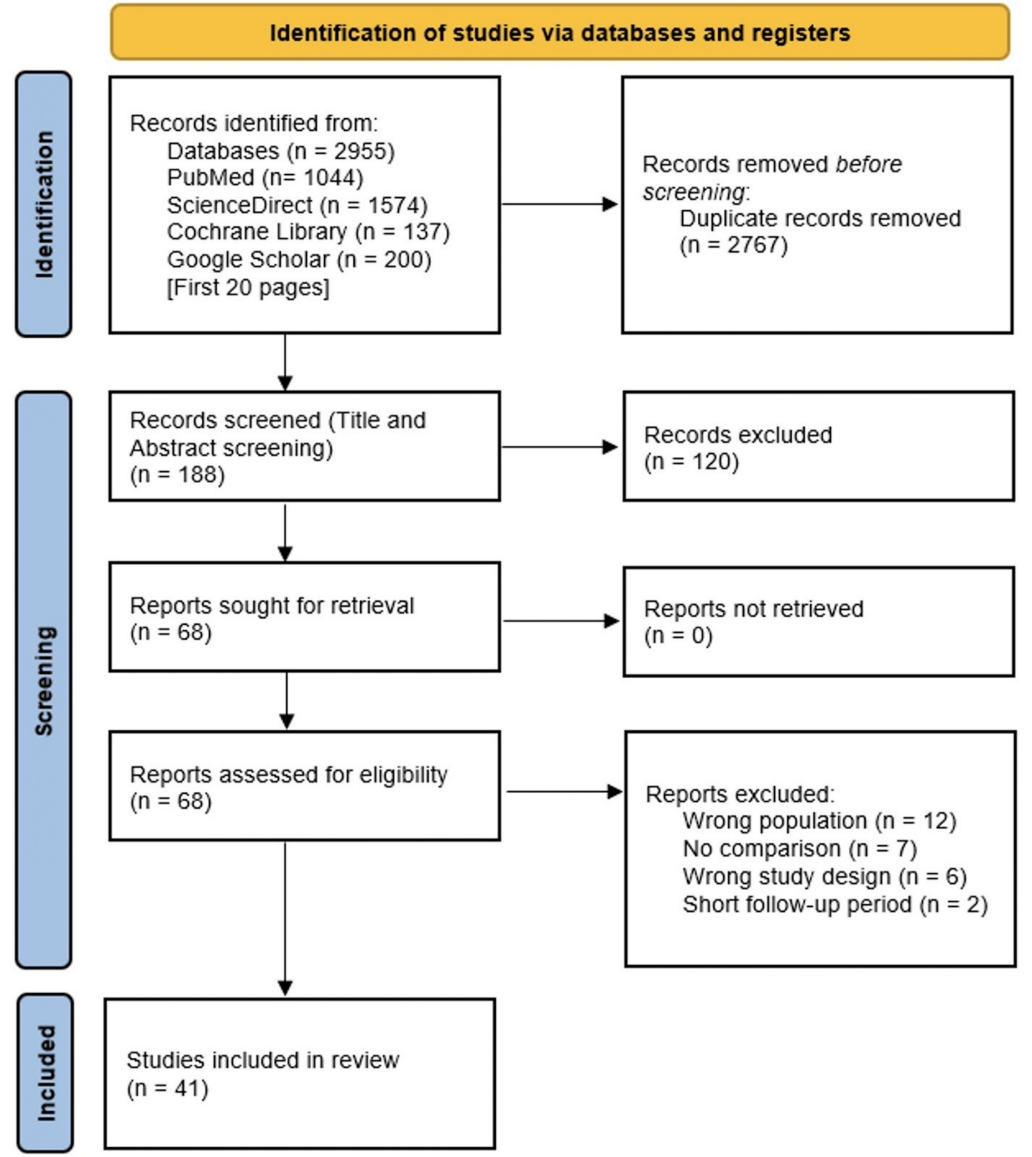
PRISMA flowchart showing the study selection process PRISMA flow diagram showing the selection process for studies included in the systematic review and meta-analysis. A total of 2,955 records were identified through database searches (PubMed, ScienceDirect, Cochrane Library, and Google Scholar). After removing 2,767 duplicates, 188 records underwent title and abstract screening. Following exclusion of 120 records, 68 full-text articles were assessed for eligibility. Of these, 27 were excluded due to wrong population (n = 12), absence of comparison group (n = 7), unsuitable study design (n = 6), or inadequate follow-up (n = 2). Ultimately, 41 studies were included in the final review.

The initial database search yielded a total of 2,955 articles, and 2,767 duplicates were removed. The remaining 188 articles were subjected to a title and abstract screening, with 120 of them being excluded due to topic irrelevancy. Most of the articles were excluded because they had no comparison between the SBRT and surgery groups. The remaining 68 articles were subjected to a full-text review, but only 41 of them were eligible for inclusion. Figure 1 depicts the flow diagram. 

Study Characteristics

A total of 41 studies were included: 38 retrospective studies employing propensity score matching, two standard retrospective studies, and one randomized controlled trial. These studies comprised 88,228 patients diagnosed with early-stage NSCLC, of whom 58,366 underwent surgical resection and 29,862 received SBRT. Comparisons between SBRT and lobectomy, mixed surgical procedures, and sublobar resections were reported in 12, five, and seven studies, respectively. In addition, four studies provided separate comparisons between SBRT and both lobectomy and sublobar resection. The SBRT intervention varied, with total doses ranging from 45 to 60 Gy delivered over three to 12 fractions. Details regarding study design, patient numbers, and clinical and treatment characteristics are presented in Table 1.

**Table 1:** Characteristics of the studies included in the review US: United States; SBRT: stereotactic body radiotherapy; RS: retrospective; fx: fraction; SL: sublobar resection; y: year; OS: overall survival; LC: local control; CSS: cancer-specific survival; PSM: propensity score matching; L: lobectomy; NR: not reported; RC: regional control; DC: disease control; PFS: progression-free survival; LRC: locoregional control; RFS: relapse-free survival; RCT: randomized controlled trial; BED: biological effective dose.

Author (s)	Year	Country	Study design	Clinical stage	No. of patients (Male %)		Radiation dose (Gy)	Type of surgery	Outcome	Follow-up (Months)
					SBRT	Surgery				
de Ruiter et al. [22]	2024	Netherlands	RS PSM	Stage 1	972 (54.1)	211 (51.8)	BED < 100 Gy	L and Mixed	1y and 5y OS and RFS	NR
Mansur et al. [23]	2024	US	RS PSM	Stage 1	130 (43.1)	130 (44.6)	NR	Mixed	1y and 5y OS	NR
Yun et al. [24]	2023	South Korea	RS PSM	Stage 1	43 (69.8)	339 (53.7)	60 Gy; 3-4 fx	SL	1y and 3y OS and RFS	42
de Ruiter et al. [25]	2022	Netherlands	RS PSM	Stage 1	241 (54.8)	356 (51.1)	NR	L and Mixed	1y, 3y and 5y PFS, OS, and CSS	12 and 24
Kishi et al. [26]	2022	Japan	RS PSM	Stage 1	150 (75.3)	407 (54.8)-L 101 (64.4)-SL	48 Gy; 4 fx, 60 Gy; 8 fx	L and SL	1y and 5y OS and RFS	NR
Littau et al. [27]	2022	US	RS PSM	Stage 1	5465 (42)	20498 (39.2)	NR	Mixed	1y and 5y OS	NR
Razi et al. [28]	2021	US	RS PSM	T1/2aN0M0	286 (40.6)	8964 (44.3)	50 Gy; 4 fx	L	1y, 3y, and 5y OS	31 and 42
Dong et al. [29]	2020	China	RS PSM	T1-2N0M0	109 (73)	121 (54)	50 Gy; 4 fx, 50 Gy; 5 fx	SL	1y, 3y, and 5y OS, CSS and DFS	NR
Dong et al. [30]	2020b	China	RS PSM	T1-2N0M0	52 (60)	52 (60)	50 Gy; 4 fx, 50 Gy; 5 fx	L and Mixed	1y, 3y, and 5y OS, CSS, and DFS	44
Wu et al. [31]	2020	China	RS PSM	T1/2aN0M0	9967	9967	NR	SL	1y, 2y, 3y, and 5y OS	NR
Kastelijn et al. [32]	2019	Netherlands	RS PSM	T1-3N0M0	53 (36)	175 (62)	54 Gy; 3 fx ,60 Gy; 5 fx, 60 Gy; 8 fx	Mixed and L	1y, 3y, and 5y OS, CSS, LRC, and RFS	39
Eba et al. [33]	2019	Japan	RS PSM	T1N0M0	21 (50)	21 (49.3)	48 Gy;4 fx	L	1y, 3y, and 5y OS	NR
Lin et al. [34]	2018	China	RS PSM	T1/2aN0MO	45 (60)	45 (60)	NR	L	1y and 3y OS, CSS, and LRC.	NR
Puri et al. [35]	2018	US	RS PSM	T1/2N0M0	5355	5355	54 Gy; 3 fx	L	3y and 5y OS and CSS	17 and 28
Verstegen et al. [15]	2018	Netherlands	RS PSM	T1-3N0M0	64 (57.8)	64 (56.3)	54-60 Gy; 3-12 fx	L	1y and 3y OS, LC, RC, PFS, and DC.	16 and 30
Bryant et al. [36]	2017	US	RS	T1/2aN0M0	499 (97)	L-2,986 (96) & SL-634 (96)	BED > 100	L and SL	1y and 3y OS and CSS.	31
Dong et al. [37]	2017	China	RS PSM	T1/2aN0MO	66 (65.2)	66 (63.6)	BED > 100	Mixed	1y and 3y OS, CSS, and LRC.	NR
Miyazaki et al. [38]	2016	Japan	RS PSM	T1N0M0	27 (63)	27 (67)	48 Gy; 4 fx, 60 Gy; 10 fx	Mixed	1y, 3y and 5y OS and CSS.	NR
Albano et al. [39]	2016	US	RS PSM	T1-3N0M0	48	64	48 Gy; 4 fx	L	1y, 3y and 5y OS and LC.	NR
Cornwell et al. [40]	2016	US	RS PSM	T1/2aN0M0	37 (97.3)	37 (97.3)	BED > 100	L	1y, 3y, and 5y OS, CSS, LRC, and RFS.	44
Ezer et al. [41]	2015	Canada	RS PSM	T1/2N0M0	362 (35)	1881 (43)	BED > 100, Gy10	SL	1y and 3y OS and CSS.	27 and 38
Mokhles et al. [42]	2015	Netherlands	RS PSM	T1/2aN0M0	73 (58)	73 (60)	54-60Gy, 3-8fx	L	1y, 3y, and 5y OS, CSS, LRC, and RFS.	36 and 49
Smith et al. [43]	2015	US	RS PSM	T1/2aN0M0	300 (42) 243 (41)	L-300 (38) SL-243 (39)	NR	L and SL	1y and 3y OS and CSS.	44 and 49
Van den Berg et al. [44]	2015	Netherlands	RS APC	T1/2aN0M0	197 (73)	143 (67)	60 Gy; 3-8 fx	Mixed	1y, 3y, and 5y OS, CSS, LRC, and RFS.	60
Wang et al. [45]	2015	China	RS PSM	T1N0M0	35 (94.3)	35 (94.3)	54-60 Gy; 3-8 fx	L and Mixed	1y, 3y, and 5y OS, CSS, LRC, and RFS.	NR
Paul et al. [46]	2015	US	RS PSM	T1/2N0M0	201	201	NR	SL	1y, 3y, and 5y OS, CSS, LRC, and RFS.	35
Rosen et al. [47]	2015	US	RS PSM	T1/2aN0M0	1781 (43)	1781 (44)	BED > 100	L	1y, 3y, and 5y OS.	29 and 32
Yerokun et al. [48]	2015	US	RS PSM	T1N0M0	1584	1584	NR	SL	1y, 3y, and 5y OS.	30
Chang et al. [49]	2014	Netherlands	RCT	T1/2aN0M0	31 (45)	27 (41)	54-60 Gy; 3-5 fx	L	1y and 3y OS, LC, RC, PFS, and DC.	NR
Boyer et al. [50]	2014	US	RS PSM	Stage 1	400	400	NR	L	2y, 4y, 6y, 8y, and 10y OS and CSS	NR
Shirvani et al. [51]	2014	US	RS PSM	T1/2bN0M0	251 53	251-L 53-SL	NR	L and SL	1y, 2y, and 3y OS and CSS	NR
Hamaji et al. [52]	2014	Japan	RS PSM	T1/2aN0M0	41 (75.6)	41 (78)	48 Gy; 4 fx	L	1y, 3y, and 5y OS, CSS, LC, RC, PFS and DC.	41 and 54
Robinson et al. [53]	2013	Canada	RS PSM	T1-3N0M0	76 (55.3)	76 (48.7)	54 Gy; 3 fx, 50 Gy; 5 fx, 45 Gy; 3 fx	L and Mixed	1y and 3y OS, CSS, LC, RC, and DC.	50
Varlotto et al. [54]	2013	US	RS PSM	T1/2N0M0	77	77	48-60 Gy, 3-5 fx	L and Mixed	1y, 3y, and 5y OS, LC, RC, and DC.	19 and 30
Crabtree et al. [55]	2013	US	RS PSM	T1/2aN0M0	56 (51.8)	56 (57.1)	45 Gy; 5 fx, 48 Gy; 4 fx, 50 Gy; 5 fx, 60 Gy; 5 fx	L and Mixed	1y and 3y OS, LC, RC, PFS, and DC.	23 and 50
Shirvani et al. [56]	2012	US	RS PSM	T1/2N0M0	99 (59.6) 112 (62.5)	99 (65.7)-L 112 (58.9)-SL	NR	L and SL	OS, CSS, LC, RC, and DC.	38
Palma et al. [57]	2011	Netherlands	RS PSM	T1/2N0M0	60 (67)	60 (67)	60 Gy; 3-8 fx	L and Mixed	1y and 3y OS.	43
Grills et al. [58]	2010	US	RS	T1/2aN0M0	58 (40)	69 (38)	48 Gy; 4 fx, 60 Gy; 5 fx	SL	1y and 3y OS, LC, and CSS.	30
Crabtree et al. [59]	2010	US	RS PSM	T1/2aN0M0	57	57	54 Gy; 3 fx	L and Mixed	1y, 3y, and 5y OS, LC, and CSS.	19 and 31

Results of Quality Appraisal

The quality of the 41 included studies was assessed using the Newcastle-Ottawa Scale. All studies achieved scores ranging from 6 to 8, indicating high methodological quality and a low risk of bias (Appendix 2). This consistent quality across studies strengthens the reliability of the overall findings in the review.

Overall Survival

**Figure 2:**
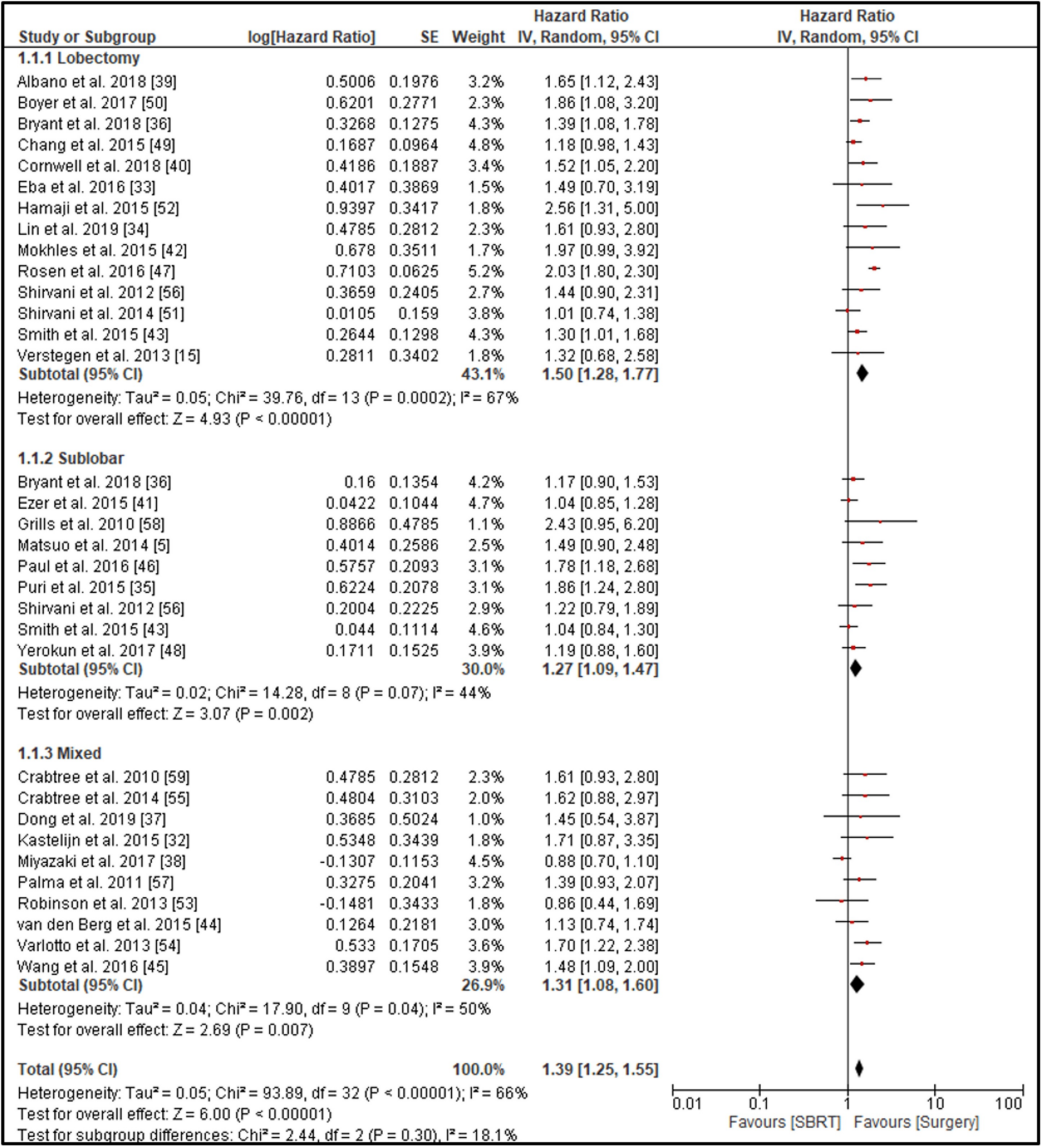
Forest plot of three-year overall survival comparing lobectomy, sublobar resection, and mixed surgery vs. SBRT Forest plot comparing overall survival (OS) between surgery and SBRT in patients with early-stage non-small cell lung cancer (NSCLC). The plot presents hazard ratios (HRs) with 95% confidence intervals (CIs) for individual studies, stratified by surgical modality: lobectomy (13 studies), sublobar resection (8 studies), and mixed surgical approaches (11 studies). Pooled results show a statistically significant OS benefit in favor of surgery (HR = 1.39; 95% CI: 1.25–1.55; p < 0.00001), with lobectomy providing the greatest survival advantage (HR = 1.50; 95% CI: 1.28–1.77). Subgroup heterogeneity and statistical significance are indicated. An HR >1 favors surgery, while HR <1 favors SBRT.

A total of 33 studies were included in the meta-analysis of overall survival. When comparing SBRT to surgery for early-stage NSCLC, the pooled HR for three-year OS significantly favored surgical resection, with an HR of 1.39 (95% CI: 1.25-1.55; p < 0.00001) (Figure 2).

**Figure 3:**
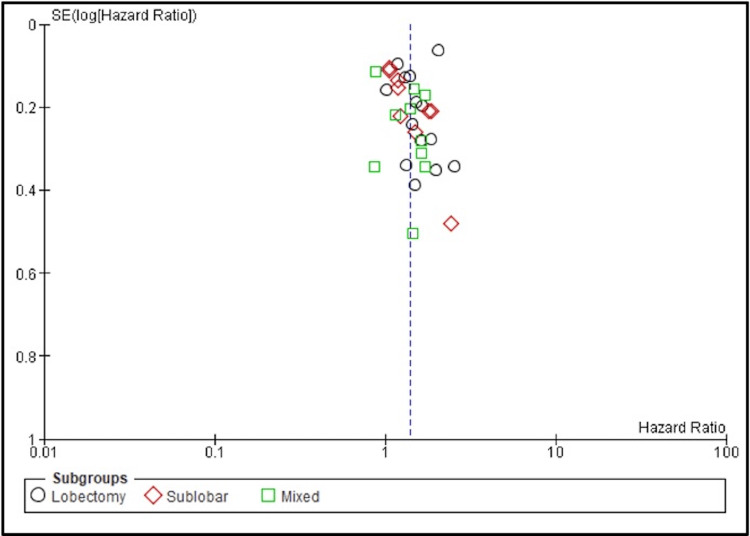
Publication bias Funnel plot assessing publication bias among studies comparing overall survival (OS) between stereotactic body radiotherapy (SBRT) and surgery in early-stage non-small cell lung cancer (NSCLC). Each point represents an individual study, categorized by surgical subgroup: lobectomy (black circles), sublobar resection (red diamonds), and mixed (green squares). The x-axis displays the hazard ratio on a logarithmic scale, and the y-axis shows the standard error of the log hazard ratio. Symmetry around the vertical reference line (log HR = 0) suggests minimal publication bias.

Moderate heterogeneity was observed among the studies (I² = 66%) (Figure 3).

Subgroup analyses based on the type of surgical procedure revealed consistent findings. Fourteen studies comparing SBRT to lobectomy showed a significantly higher mortality risk with SBRT (HR = 1.50; 95% CI: 1.28-1.77; I² = 67%; p < 0.00001). The comparison with sublobar resection across several studies demonstrated a significant survival advantage for surgery (HR = 1.27; 95% CI: 1.09-1.47; I² = 44%; p = 0.002). In studies where SBRT was compared to mixed surgical procedures, the pooled estimate continued to favor surgery (HR = 1.39; 95% CI: 1.08-1.60; I² = 50%; p = 0.007). These findings indicate that surgical resection, particularly lobectomy, is associated with significantly improved three-year overall survival compared to SBRT in patients with early-stage NSCLC.

Cancer-Specific Survival

**Figure 4:**
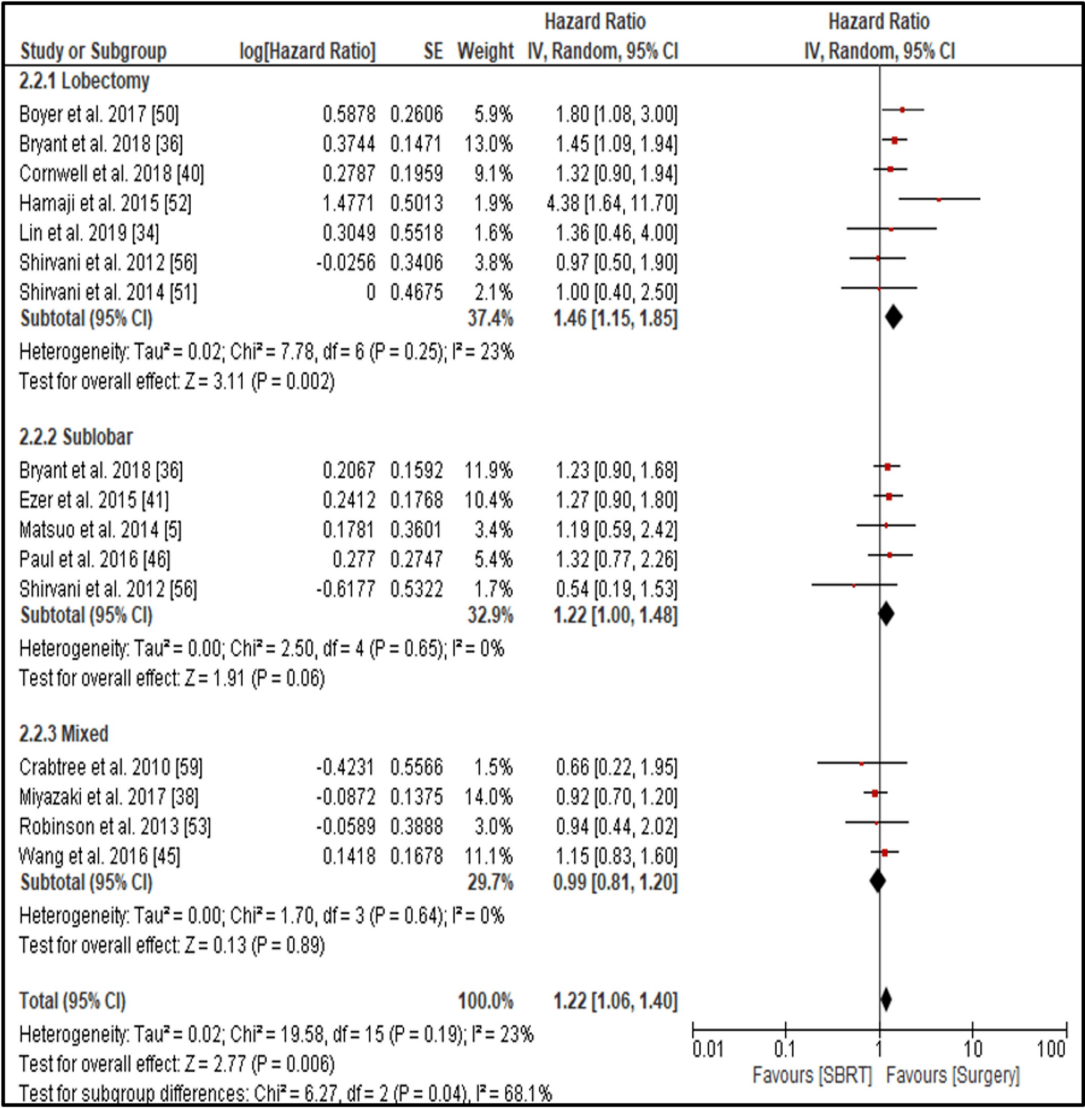
Forest plot comparing cancer-cpecific survival (CSS) between surgery and SBRT in early-stage NSCLC Forest plot showing the hazard ratios (HRs) with 95% confidence intervals (CIs) for cancer-specific survival (CSS) from studies comparing surgery and stereotactic body radiotherapy (SBRT) in early-stage non-small cell lung cancer (NSCLC). Results are stratified by surgical technique: lobectomy, sublobar resection, and mixed approaches. Lobectomy was associated with a significant CSS benefit over SBRT (HR = 1.46; 95% CI: 1.15–1.85; p = 0.002), while sublobar resection showed a borderline benefit (HR = 1.22; 95% CI: 1.00–1.48; p = 0.06). Mixed surgical approaches did not demonstrate a significant advantage (HR = 0.99; 95% CI: 0.81–1.20; p = 0.89). The overall pooled HR indicated a significant CSS benefit for surgery (HR = 1.22; 95% CI: 1.06–1.40; p = 0.006). An HR >1 favors surgery.

The pooled three-year CSS significantly favored surgery over SBRT (HR = 1.22, 95% CI: 1.06-1.40; p = 0.006) (Figure 4).

**Figure 5:**
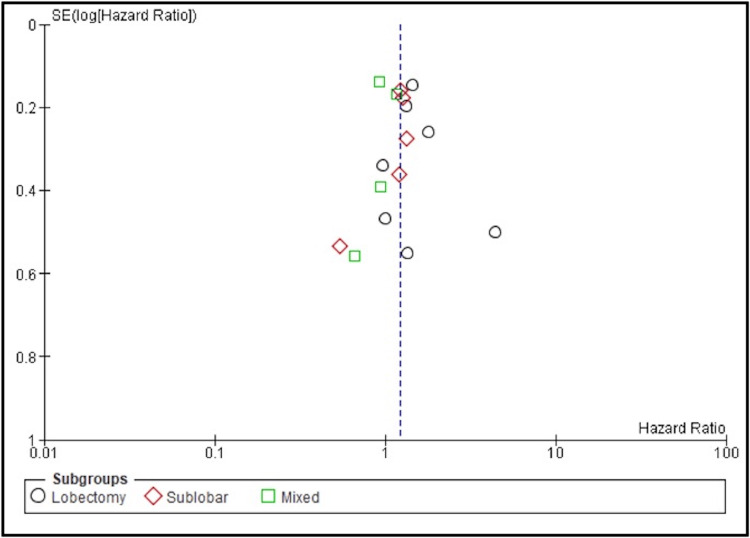
Funnel plot for cancer-specific survival (CSS) in SBRT vs. surgery comparisons Funnel plot evaluating publication bias among studies comparing cancer-specific survival (CSS) between stereotactic body radiotherapy (SBRT) and surgical resection in early-stage non-small cell lung cancer (NSCLC). Studies are categorized by surgical subgroup: lobectomy (black circles), sublobar resection (red diamonds), and mixed approaches (green squares). The x-axis displays the hazard ratio (HR) on a logarithmic scale, and the y-axis represents the standard error of the log HR. The vertical dashed line indicates the line of no effect (HR = 1). The relative symmetry suggests low risk of publication bias.

Heterogeneity across the studies was low (I² = 23%) (Figure 5).

Subgroup analysis further reinforced this trend. In seven studies comparing SBRT with lobectomy, the pooled HR was 1.46 (95% CI: 1.15-1.85; p = 0.002; I² = 23%), a statistically significant result indicating better CSS with lobectomy. Comparisons with sublobar resection yielded an HR of 1.22 (95% CI: 1.00-1.48; p = 0.06; I² = 0%), which did not reach statistical significance. Similarly, in studies comparing SBRT with mixed surgical procedures, the result was non-significant (HR = 0.99; 95% CI: 0.81-1.20; p = 0.89; I² = 0%). These findings indicate that lobectomy is associated with significantly better CSS compared to SBRT, while sublobar and mixed resections do not show a statistically significant advantage.

Local Control

**Figure 6:**
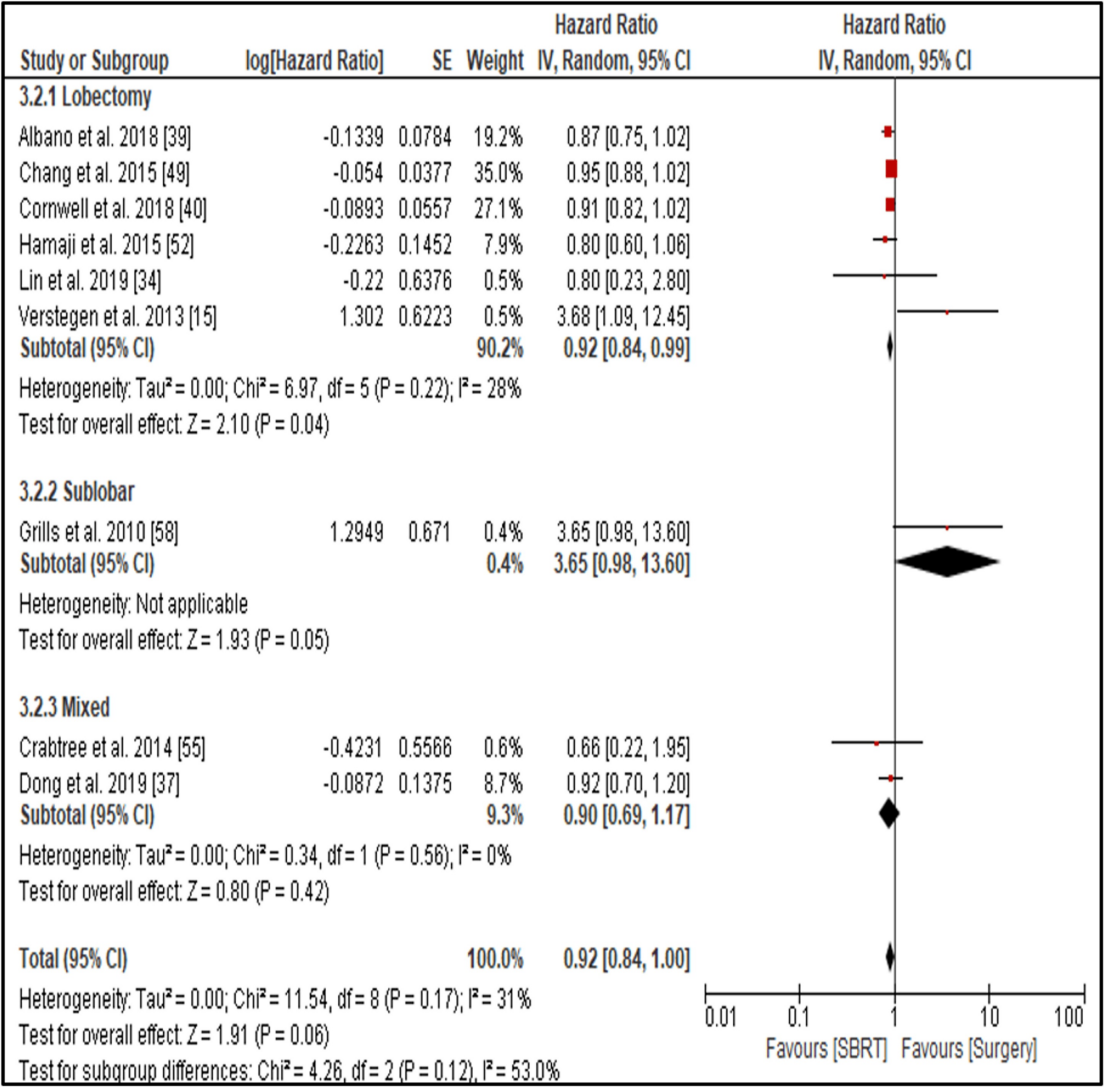
Forest plot comparing local control (LC) between surgery and SBRT in early-stage NSCLC Forest plot displaying hazard ratios (HRs) with 95% confidence intervals (CIs) for local control (LC) outcomes in studies comparing surgery and stereotactic body radiotherapy (SBRT) for early-stage non-small cell lung cancer (NSCLC). Subgroup analysis by surgical approach shows that lobectomy significantly favored surgery over SBRT (HR = 0.92; 95% CI: 0.84–0.99; p = 0.04). Sublobar resection showed a non-significant trend toward poorer local control (HR = 3.65; 95% CI: 0.98–13.60; p = 0.05), and mixed surgical groups did not show a significant difference (HR = 0.90; 95% CI: 0.69–1.17; p = 0.42). Overall, surgery demonstrated a marginally significant benefit in local tumor control compared to SBRT (HR = 0.92; 95% CI: 0.84–1.00; p = 0.06). An HR <1 favors surgery.

**Figure 7:**
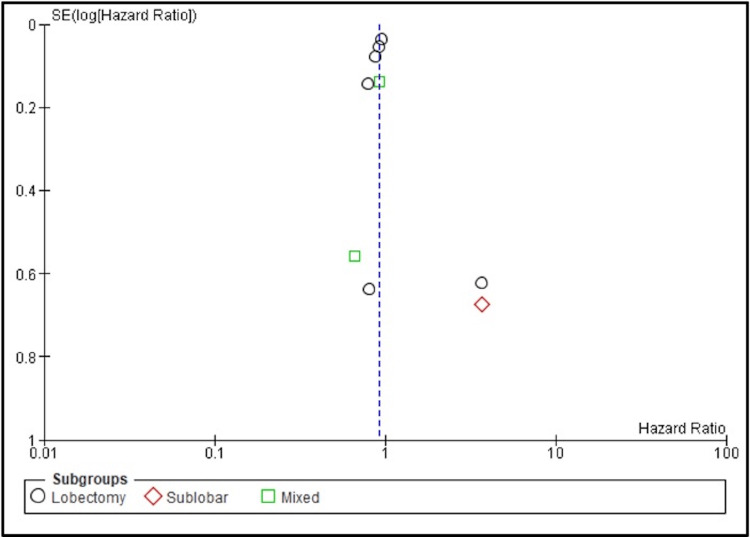
Funnel plot for local control (LC) in SBRT vs. surgery comparisons Funnel plot evaluating potential publication bias in studies comparing local control (LC) between stereotactic body radiotherapy (SBRT) and surgical resection in early-stage non-small cell lung cancer (NSCLC). Each point represents an individual study and is categorized by surgical subgroup: lobectomy (black circles), sublobar resection (red diamonds), and mixed approaches (green squares). The x-axis displays hazard ratios (HRs) on a logarithmic scale, and the y-axis shows the standard error of the log HR. The vertical dashed line represents the line of no effect (HR = 1). Limited asymmetry, indicating low risk of publication bias.

The comparison between SBRT and surgery yielded a pooled HR of 0.92 (95% CI: 0.84-1.00; p = 0.06; I² = 31%) (Figures 6, 7), indicating no statistically significant difference in three-year LC between the two treatments.

However, subgroup analysis revealed some important findings. Six studies comparing SBRT to lobectomy demonstrated a statistically significant advantage for lobectomy, with a pooled HR of 0.92 (95% CI: 0.84-0.99; p = 0.04; I² = 28%). The sublobar subgroup, when compared to SBRT, showed a trend toward worse outcomes with a high HR of 3.65 (95% CI: 0.98-13.00; p = 0.05), although this did not reach statistical significance, likely due to wide confidence intervals and limited sample size. Comparisons involving mixed surgical procedures also did not show a significant difference (HR = 0.90; 95% CI: 0.69-1.17; p = 0.42; I² = 0%). These results suggest that lobectomy may provide a statistically significant local control benefit over SBRT.

Discussion

This updated meta-analysis provides comprehensive evidence comparing the efficacy of SBRT versus surgical resection in the management of early-stage NSCLC. Our findings reveal that surgical intervention is associated with significantly improved outcomes in terms of OS and CSS. The pooled HR for three-year OS favored surgery over SBRT (HR = 1.39; 95% CI: 1.25-1.55; p < 0.00001), with similar trends observed in subgroup analyses for lobectomy (HR = 1.50), sublobar resection (HR = 1.27), and mixed surgical procedures (HR = 1.39). Cancer-specific survival also favored surgery (HR = 1.22; 95% CI: 1.06-1.40; p = 0.006), with lobectomy showing a particularly notable advantage (HR = 1.46; p = 0.002). While local control (LC) outcomes were generally comparable between groups, lobectomy demonstrated a modest but significant advantage over SBRT (HR = 0.92; p = 0.04). These findings reinforced the survival benefit of surgery in operable patients, while also recognizing the role of SBRT as a viable alternative for selected patients.

Our results are largely consistent with earlier reviews and meta-analyses. A 2017 meta-analysis by Wen et al. found surgery to be superior in overall survival and cancer-specific survival, supporting our observation of improved OS with surgical intervention [17]. Similarly, Cao et al. reported that while SBRT was associated with fewer perioperative complications, long-term survival favored surgery in patients with good performance status [60]. Our findings update and extend these observations by incorporating recent studies with improved adjustment for confounding variables, including 38 propensity score-matched studies and one randomized trial. Notably, the pooled HRs in our study reflect slightly lower effect estimates than some earlier reviews, possibly due to the inclusion of more contemporary SBRT protocols and better patient selection in newer cohorts. In addition, our analysis of local control shows minimal differences between modalities, which aligns with the growing recognition of SBRT’s ability to achieve excellent tumor control in peripheral tumors, as highlighted by Chang et al. in the STARS and ROSEL trials [49]. However, the apparent survival benefit with lobectomy underscores the potential impact of comprehensive mediastinal staging and nodal clearance, which remain limitations of SBRT.

Surgical resection offers the advantage of complete tumor removal with pathological staging, enabling more accurate assessment of nodal involvement and potentially guiding adjuvant therapy. This may partly explain the superior survival outcomes observed in our analysis. In contrast, SBRT provides a non-invasive alternative with minimal recovery time, fewer perioperative risks, and high local control rates, making it particularly valuable for medically inoperable patients or those with significant comorbidities. The evolving precision and dosimetric improvements in SBRT have further narrowed the gap in local efficacy between the two modalities.

Study Limitations

This review has several limitations. The majority of included studies were retrospective in design, which introduces inherent risks of selection bias - patients undergoing surgery are typically healthier and more likely to have fewer comorbidities than those selected for SBRT, potentially skewing survival outcomes in favor of surgery. While the use of propensity score matching in many of these studies strengthens internal validity by attempting to balance measured confounders, it cannot account for unmeasured variables such as frailty or pulmonary function. Only one RCT was included, underscoring the ongoing lack of high-level evidence directly comparing SBRT and surgery in operable patients. In addition, variation in SBRT dosing regimens across studies (ranging from 45 to 60 Gy in 3 to 12 fractions) may have influenced treatment efficacy, particularly in relation to tumor location and size, and may limit the generalizability of pooled estimates.

Future Directions

The findings of this meta-analysis suggest that surgical resection, particularly lobectomy, remains associated with superior overall and cancer-specific survival compared to SBRT in patients with early-stage NSCLC, supporting its continued role as the standard of care in operable individuals. However, the comparable local control and favorable safety profile of SBRT reinforce its value as a non-invasive alternative, especially for patients who are medically inoperable or prefer to avoid surgery. These results highlight the importance of individualized, multidisciplinary treatment planning that considers patient comorbidities, performance status, and preferences. Looking forward, high-quality RCTs are urgently needed to definitively compare SBRT and surgery in operable populations, ideally stratifying by tumor size, location, and biological behavior. Future research should also explore optimal SBRT dosing strategies, integration with systemic therapies, and long-term outcomes including quality of life and functional preservation.

## Conclusions

The use of SBRT has emerged as a valuable and less invasive treatment option for patients with early-stage NSCLC, particularly those who are not surgical candidates. However, our meta-analysis indicates that surgical resection - especially lobectomy - continues to be associated with superior OS and CSS compared to SBRT, despite similar local control rates. These findings reinforce the role of surgery as the standard of care in operable patients while supporting SBRT as an effective alternative for high-risk or medically inoperable individuals. Continued comparative research, particularly randomized trials, is essential to refine patient selection criteria and optimize treatment strategies.

## Appendices

Appendix 1

**Table 2:** Search strategy

Database	Search string
PubMed	("non-small cell lung carcinoma" OR "non-small cell lung cancer" OR "lung adenocarcinoma" OR "lung squamous cell carcinoma" OR "large cell lung cancer") AND ("surgery" OR "lobectomy" OR "sublobar resection" OR "limited resection" OR "sublobectomy" OR "segmentectomy" OR "wedge resection") AND ("stereotactic ablative radiotherapy" OR "stereotactic body radiotherapy" OR "SBRT" OR "SABR")
ScienceDirect	("non-small cell lung cancer" OR "non-small cell lung carcinoma" OR "lung adenocarcinoma") AND (surgery OR lobectomy) AND ("stereotactic body radiotherapy" OR "stereotactic ablative radiotherapy" OR SBRT OR SABR)
Cochrane Library	("non-small cell lung cancer" OR "lung adenocarcinoma" OR "lung squamous cell carcinoma") AND (surgery OR lobectomy) AND (SBRT OR SABR)
Google Scholar	(“non-small cell lung carcinoma” OR “non-small cell lung cancer” OR “non-small cell lung neoplasms” OR “lung adenocarcinoma” OR “lung squamous cell carcinoma” OR “large cell lung cancer”) AND (“surgery” OR “lobectomy” OR “sublobar resection” OR “limited resection” OR “sublobectomy” OR “segmentectomy” OR “wedge resection”) AND (“stereotactic ablative radiotherapy” OR “stereotactic body radiotherapy” OR “SBRT” OR “SABR”)

Appendix 2

**Table 3:** Quality appraisal Item 1: Representativeness of the exposed cohort, Item 2: Selection of the non-exposed cohort, Item 3: Ascertainment of exposure, Item 4: Demonstration that outcome of interest was not present at start of study, Item 5: Comparability of cohorts on the basis of the design or analysis, Item 6: Assessment of outcome, Item 7: Was follow-up long enough for outcomes to occur, Item 8: Adequacy of follow-up of cohorts Interpretation: 1-3 = low quality, 4-5 = moderate quality, 6-8 = high quality

Study	Item 1	Item 2	Item 3	Item 4	Item 5	Item 6	Item 7	Item 8	Total
de Ruiter et al. [22]	1	1	1	1	1	1	1	1	8
Mansur et al. [23]	1	1	1	1	1	1	1	1	8
Yun et al. [24]	1	1	1	1	1	1	1	1	8
de Ruiter et al. [25]	1	1	1	1	0	1	1	1	7
Kishi et al. [26]	1	1	1	1	1	1	1	1	8
Littau et al. [27]	1	1	1	1	1	1	1	1	8
Razi et al. [28]	1	1	1	1	1	1	1	1	8
Dong et al. [29]	1	1	1	1	0	1	1	0	6
Dong et al. [30]	1	1	1	1	1	0	1	1	7
Wu et al. [31]	1	1	1	1	1	1	1	1	8
Kastelijn et al. [32]	1	1	1	1	1	1	1	1	8
Eba et al. [33]	1	1	1	1	0	1	1	1	7
Lin et al. [34]	1	1	1	1	1	0	1	1	7
Puri et al. [35]	1	1	1	1	1	1	1	1	8
Verstegen et al. [15]	1	1	1	1	1	1	1	1	8
Bryant et al. [36]	1	1	1	1	0	1	1	1	7
Dong et al. [37]	1	1	1	1	1	1	1	1	8
Miyazaki et al. [38]	1	1	1	1	1	0	1	0	6
Albano et al. [39]	1	1	1	1	1	1	1	1	8
Cornwell et al. [40]	1	1	1	1	1	1	1	1	8
Ezer et al. [41]	1	1	1	1	0	1	1	1	7
Mokhles et al. [42]	1	1	1	1	1	1	1	1	8
Smith et al. [43]	1	1	1	1	1	1	1	1	8
Van den Berg et al. [44]	1	1	1	1	1	1	1	1	8
Wang et al. [45]	1	1	1	1	0	0	1	1	6
Paul et al. [46]	1	1	1	1	1	1	1	1	8
Rosen et al. [47]	1	1	1	1	1	0	1	1	7
Yerokun et al. [48]	1	1	1	1	0	1	1	1	7
Chang et al. [49]	1	1	1	1	1	1	1	1	8
Boyer et al. [50]	1	1	1	1	1	1	1	0	7
Shirvani et al. [51]	1	1	1	1	1	0	1	1	7
Hamaji et al. [52]	1	1	1	1	1	1	1	1	8
Robinson et al. [53]	1	1	1	1	1	1	1	0	7
Varlotto et al. [54]	1	1	1	1	0	1	1	1	7
Crabtree et al. [55]	1	1	1	1	1	1	1	1	8
Shirvani et al. [56]	1	1	1	1	1	1	1	0	7
Palma et al. [57]	1	1	1	1	1	1	1	1	8
Grills et al. [58]	1	1	1	1	1	1	1	1	8
Crabtree et al. [59]	1	1	1	1	1	1	1	1	8
